# Acute caffeine supplementation enhances several aspects of shot put performance in trained athletes

**DOI:** 10.1080/15502783.2022.2096415

**Published:** 2022-07-06

**Authors:** Verónica Giráldez-Costas, Millán Aguilar-Navarro, Jaime González-García, Juan Del Coso, Juan José Salinero

**Affiliations:** a Camilo José Cela University, Exercise Physiology Laboratory, Madrid, Spain; b Universidad Francisco de Vitoria, Faculty of Health Science, Madrid, Spain; c Rey Juan Carlos University, Centre for Sport Studies, Fuenlabrada, Spain; d Castilla-La Mancha University, Faculty of Sport Sciences. Sport Training Laboratory (GIRD), Toledo, Spain

**Keywords:** Ergogenic aids, athletics, elite athlete, throwing, vertical jump

## Abstract

The aim of this investigation was to determine the effect of a moderate dose of caffeine (3 mg/kg/b.m.) on muscular power and strength and shot put performance in trained athletes. Methods. Thirteen shot putters (eight men and five women) participated in a double-blind, placebo-controlled, randomized experiment. In two different trials, participants ingested either 3 mg/kg/b.m. of caffeine or a placebo. Forty-five min after substance ingestion, athletes performed a handgrip dynamometry test, a countermovement jump (CMJ), a squat jump (SJ), and a maximum-velocity push-up. The athletes also performed three types of throws: a backwards throw, a standing shot put and a complete shot put. Results. In comparison with the placebo, caffeine ingestion increased CMJ height (32.25 ± 7.26 *vs*. 33.83 ± 7.72 cm, respectively; effect size (ES) = 0.82, p = 0.012; +5.0%;) and SJ height (29.93 ± 7.88 *vs*. 31.40 ± 7.16 cm; ES = 0.63, p = 0.042; +6.4%) and distance in the standing shot put (10.27 ± 1.77 m *vs*. 10.55 ± 1.94 m; ES = 0.87, p = 0.009; +2.6%). However, caffeine ingestion did not increase strength in the handgrip test, power in the ballistic push-up, or distance in the backwards throw (all p > 0.05). Shot put performance changed from 11.24 ± 2.54 to 11.35 ± . 2.57 m (ES = 0.33, p = 0.26; +1.0%), although the difference did not reach statistically significant differences. Caffeine ingestion did not increase the prevalence of side effects (nervousness, gastrointestinal problems, activeness, irritability, muscular pain, headache, and diuresis) in comparison with the placebo (p > 0.05). Conclusion. In summary, caffeine ingestion with a dose equivalent to 3 mg/kg/b.m. elicited moderate improvements in several aspects of physical performance in trained shot putters but with a small effect on distance in a complete shot put.

## 1. Introduction

Caffeine is widely consumed in the sports context. According to the caffeine concentration in urine samples obtained after competition for anti-doping purposes, three out of four elite athletes had consumed caffeine before or during competition [1]. Athletics was among the sports with the highest urine caffeine concentration and the concentration of caffeine in the sample of athletes has doubled since the elimination of caffeine from the list of banned substances [1]. The main cause of this high use of caffeine in Athletics is likely due to the strong evidence of the ergogenic effect of caffeine on a wide spectrum of exercise situations. Supplementation with caffeine prior to exercise (at a dosage of 3–6 mg per kg of body mass; mg/kg/b.m.), acutely improves various aspects of physical performance [2–7], including aerobic [3,5] and anaerobic [8,9] activities, and strength and power [3,10,11]. Strangely enough, few studies have analyzed the translation of these caffeine-induced ergogenic effects on physical variables to specific benefits in complex athletics disciplines, such as throwing events.

Athletics is a complex sport because it includes a variety of disciplines with very different physical and physiological requirements to achieve high performance. Overall, the disciplines of Athletics can be grouped into races, jumping and throwing events. While the International Association of Athletics Federations Consensus Statement recommends caffeine as an ergogenic aid for different event groups in Athletics [12], the truth is that running disciplines with a more aerobic-like nature, such as middle- and long-distance races, have received more attention in caffeine research [13–16] than jumping and throwing events. One recent investigation concluded that acute caffeine ingestion improves high-jump but not long-jump performance [17]. Similarly, to the authors’ knowledge, there is only one study on the effects of caffeine intake in throwing events [18]. Bellar et al. [18] assessed the efficacy of a low-dose of caffeine (*i.e*. 100 mg) for an early morning performance in trained college shot-putters. These authors found that caffeine produced better shot put performance over the course of six attempts. However, shot put performance during official competitions is not assessed by mean distance over six attempts but through the best distance obtained within the six attempts. Unfortunately, Bellar et al.’s investigation [18] did not present the effect of caffeine on the best throw of the six attempts and it is impossible to ascertain with their data the real impact of caffeine on shot put performance. Additionally, in Bellar’s investigation [18], the dose of caffeine employed was not individualized by body mass and it was, on average, 0.9 mg/kg/b.m., which is well below the general recommendations for caffeine supplementation in sport. In fact, the International Olympic Committee [19] and the International Society of Sports Nutrition [3] recommend the use of at least 3 to 6 mg/kg/b.m. of caffeine to enhance physical performance and, therefore, it is unknown if caffeine can enhance shot put performance when used in the dose recommended. Several systematic reviews argued that these moderate doses of caffeine improve one-repetition maximum, isometric, and isokinetic strength, rate of force development as well as muscular endurance, velocity, and power in different resistance exercises [3,11,20]. Shot put is a highly intricate discipline because demands the performance of lower and upper body movements in sequence at peak velocity while they had been to be performed within a circle 2.135 m in diameter. Hence, strength, speed, and power are keys to better performance [21,22] but the movements have to be performed with an established technique to obtain maximal distance [23]. Previous research has shown that performance in strength tests such as bench press or half squat, and explosive tests such as Olympic-style lifts, explosive-throwing bench-press or countermovement jumps (CMJ) are related to shot put performance [22,24–27]

Hence, the aim of this investigation was to determine the effect of an individualized and moderate dose of caffeine (3 mg/kg/b.m.) on muscular power, shot put-specific performance tests and, on a complete shot put throw in trained shot putters. We hypothesized that this moderate dose of caffeine would improve neuromuscular performance in shot put-specific tests and the best distance obtained during a complete shot put.

## 2. Method

### 2.1. Participants

Thirteen trained shot putters (eight men and five women) volunteered to participate in this investigation. An *a priori* sample size estimation revealed that at least 11 participants were needed to investigate the potential ergogenic effect of caffeine with an effect size of 0.996 tested with a two-tailed paired sample t-test (1 − β = 0.8; α = 0.05). This calculation was based on the effect size obtained with caffeine on standing shot put of Bellar et al.’s investigation [18] and it was performed with the G*Power (v3.1.9.6) software. Participants had a mean ± standard deviation age of 24.5 ± 10.0 years; body mass of 92.8 ± 20.6 kg; body height of 180.2 ± 8.7 cm; training experience in the discipline of 8.7 ± 6.9 years; training frequency of 8.0 ± 3.3 sessions/week; and daily caffeine consumption of 1.5 ± 0.9 mg/kg/d. All of the participants fulfilled the following inclusion criteria: a) age between 18 and 45 years old; and b) more than 1 year of shot put training experience. Participants were excluded if they reported a) sport injury within the previous two months; b) medication usage within the previous month; c) allergy to caffeine; or d) use of oral contraceptive pills, as they may interfere with caffeine pharmacokinetics [28]. Participants were asked to abstain from any form of dietary caffeine and from dietary supplement use for the duration of the study. All the participants were familiarized with the testing procedures employed in the current experiment as part of their training routines. Additionally, participants reproduced the strength and power tests in the same order and with the recovery times set for the experiment in a training session one week before the onset of the experiment. Participants were provided with informed consent forms prior to participating in the investigation in which they were informed of the experimental procedures and risks. The study was approved by the Camilo José Cela University Research Ethics Committee and was performed in accordance with the latest version of the Declaration of Helsinki.

### 2.2. Experimental design

A double-blind, placebo-controlled, randomized, and counterbalanced experimental design was used in this investigation. Each participant took part in two identical experimental trials separated by seven days to allow complete recovery, testing reproducibility, and substance wash-out. Participants acted as their own controls to produce a crossover design with repeated measures in which only the substance ingested before testing differed between the trials. In the experimental trials, the participants ingested: (a) 3 mg/kg/b.m. of caffeine (BulkPowders, 100% purity; United Kingdom); or (b) the same amount of an inert substance acting as a placebo (cellulose, Guinama, Spain). This dose was selected according to current evidence about the dose of caffeine to produce an ergogenic effect on sports performance [3,11,19]. The substances were ingested in identical unidentifiable capsules with 200 mL of water 45 minutes before the onset of the experimental testing. The trials were performed at the same time of the day and in an outdoor shot put training facility with similar ambient temperature (~14°C) and relative humidity (~40%).

### 2.3. Procedure

The participants were instructed to comply with the following conditions 24-h before each experimental trial: (i) to avoid vigorous exercise, (ii) to adopt a similar diet and drink intake, (iii) to refrain from the consumption of alcohol, caffeine, and other stimulants. On the day of the experimental trials, the study participants arrived at the facility in the morning (between 9.00 and 13.00 am) in a fed state (~3 hours after their last meal). Upon arrival, the capsule with the experimental treatment (caffeine or placebo) was provided and ingested by the participant. Then, participants rested for 25 min while they filled out a validated Food Frequency Questionnaire (FFQ) [29] to assess daily caffeine intake. Portions were used to assess the amount of food consumed and exact brands of products consumed were identified to accurately calculate caffeine consumption. Habitual caffeine intake was estimated based on the responses provided to the FFQ. Thereafter, they underwent a standardized 20-min warm-up identical to their competition routines which consisted of running, upper and lower body exercises, and several forms of throws with medicine balls. Then, the athletes started with the physical tests as follows:

### 2.4. Handgrip test

Each participant performed two maximal isometric voluntary contractions with the dominant and non-dominant hand. The highest force value out of two attempts was used for analysis, with attempts separated by 1 min of rest. Each participant performed this test with the shoulder and elbow in anatomical position, the forearm and hand in a neutral position, and handgrip force was measured with a digital dynamometer (Takei 5101, Tokyo, Japan).

### 2.5. Countermovement (CMJ) and Squat Jumps (SJ)

After 2 min of recovery, the participant performed two maximal CMJ and two maximal SJ, with the attempts separated by one minute of rest and three minutes between jump types. Participants were instructed to jump as high as possible and the highest jump for each jump type was used for analysis. Arms were placed on the hips during the jump tests to avoid the influence of the arm swing on jump height and a researcher verified that take-off and landing were performed in a correct position. Both CMJ and SJ were performed on a Force-Decks FD4000 Dual Force Platform (ForceDecks, London, United Kingdom), with a sample rate of 1,000 Hz. The highest jump was used for analysis. In each jump, peak power, and peak velocity during the concentric phase of the movement were recorded as muscular power and velocity at take off have been associated with shot put distance during the competition period [26].

### 2.6. The maximum-velocity push-up test

After 2 min of recovery, participants lay down and placed their hands on the Force-Decks FD4000 Dual Force Platform, approximately shoulder-width apart, and lowered the chest until it contacted the plates. Upon command, participants performed a maximum-velocity push-up. For this test, participants extended their arms as fast as possible to lose hand contact with the plates while the tips of the toes were always in contact with the floor. Each participant performed two maximum-velocity push-ups separated by 1 min of rest between attempts. The highest push-up was used for analysis. Peak power and peak velocity during the concentric phase of the push-up were also recorded.

### 2.7. Backwards throw, standing, and complete shot put

After the above-mentioned tests, participants went to a training shot put zone and performed an additional 5-min warm-up including different types of throws with the official shot. All throwing tests were performed on an official concrete ground outdoor circle. Participants performed the throws using an official shot for women (4 kg) and for men (7.260 kg). Each participant performed three attempts for each throw type, with the attempts separated by 3 minutes of rest and 5 minutes between throw types. The throw with the longest distance was used for the statistical analysis. Participants were encouraged to produce maximal distance throws in each attempt, and they were in a competition-like environment. The motivation was standardized and coaching was not allowed to avoid the influence of these factors on the outcomes of the experiment. First, a backwards throw was performed. For this throw, participants stood on the edge of the official concrete circle facing in the opposite direction to the direction of the throw. They then grasped the official shot with both hands, flexed their knees, and lowered the ball to place it between their knees. Starting in this position, participants extended their legs, followed by extension of their backs, and finally extension of their shoulders to throw the shot backwards over their heads, attempting to achieve maximum distance. Second, a standing shot put (i.e. without flight phase) was performed. For the standing shot put, participants put their feet about shoulder-width apart and performed only the delivery phase of the shot put, attempting to achieve maximum distance. Finally, a complete shot put was performed according to World Athletics rules (if the athlete committed a foul, they performed an additional shot put). Participants performed the complete shot put either with a glide (n = 5) or rotational style (n = 8) according to their preferences. However, the style was kept constant for all attempts and for all experimental trials. Intraclass correlation coefficient (ICC), standard error of measurement (SEM) and minimal detectable change (MDC) were calculated for the backwards throw, for the standing shot put and for the complete shot put from the two best throws in each test in the placebo condition with the following results: Backwards throw: ICC = 0.966; SEM = 0.058; MDC = 0.16 m; Standing shot put: ICC = 0.973; SEM 0.034; MDC = 0.10 m; Complete shot put: ICC = 0.984; SEM = 0.033; MDC = 0.09 m. The SEM was calculated as follows: $SEM=s\pm \sqrt{1-ICC}$. The MDC was calculated as follows: MDC = SEM*1.96*$\sqrt{2}$ [10].

At the end of each experimental trial, the athletes were required to fill out a questionnaire about their perception of muscle power. The questionnaire of perception of muscle power included a 1–10-point scale, and participants were previously informed that 1 point meant a minimal amount and 10 points meant a maximal amount. This questionnaire also included a question in which participants had to guess if they had received caffeine or placebo, to evaluate the success of the participants blinding procedure. Moreover, participants were provided with a side-effects survey to be filled out before going to sleep to determine if they had perceived any caffeine-associated side effects the hours after the capsule ingestion. This survey asked about participants’ nervousness, gastrointestinal problems, muscle pain, irritability, diuresis, headache, and activeness using a yes/no scale. These questionnaires have been previously used to assess perceived ergogenicity and the prevalence of side effects after the intake of caffeine in the sports context [30]. Participants who did not complete these questionnaires on time were eliminated from the analysis. Thus, three participants were excluded from the statistical analysis based on this criterion.

### 2.8. Statistical analysis

The study data were introduced into the statistical package JAMOVI (v. 2.2.5) and subsequently analyzed. The Shapiro–Wilk test was used to confirm the normality of each variable. All the variables presented a normal distribution and, consequently, parametric statistics were used to determine differences among trials. A paired t-test was performed to compare performance values with placebo vs. caffeine for all physical testing. Additionally, effect sizes (ES) were calculated by using the Cohen’s *d* and they were interpreted according to the following thresholds: <0.20 trivial, ≥0.20-0.59 small, ≥0.60-1.19 moderate, ≥1.20-1.99 large, and ≥2.00 very large [31]. Results are expressed as mean ± standard deviation. Data on side effects are presented as percentages to represent the proportion of athletes that reported each side effect. Differences in the prevalence of each side effect after caffeine or placebo intake were analyzed using the McNemar test. The blinding assessment was performed with Kappa index and Bang’s Index [32]. In all statistical tests, the significance level was set at p < 0.050.

## 3. Results

There were not differences between caffeine and placebo for handgrip force in either the dominant hand (48.22 ± 12.18 vs 48.38 ± 13.01 kg, ES = −0.06, p = 0.843) or the non-dominant hand (43.35 ± 11.35 vs 42.86 ± 12.25 kg, ES = 0.13, p = 0.642). The effects of caffeine on CMJ, SJ and push-up performance variables are displayed in Table 1. Caffeine significantly increased CMJ jump height (+5.0%, ES = 0.82, p = 0.012), peak power (+2.5%, ES = 0.70, p = 0.027) and peak velocity (+1.8%, ES = 0.67, p = 0.033) during the concentric phase of the jump. In the SJ, caffeine increased jump height (+6.4%, ES = 0.63, p = 0.042), and concentric peak velocity (+2.8%, ES = 0.65, p = 0.037). Nevertheless, there were no statistically significant differences in ballistic push-up between caffeine and placebo (p > 0.050) although the values for caffeine were always higher for height (+16.2%, ES = 0.56, p = 0.110), peak power (+12.7%, ES = 0.70, p = 0.055) and peak velocity (+10.6%, ES = 0.62, p = 0.082).

**Table 1.** Variables of CMJ, SJ and ballistic push up after the ingestion of 3 mg/kg/b.m. of caffeine or a placebo.

	CMJ			SJ			Push Up		
	Placebo	Caffeine	ES	Placebo	Caffeine	ES	Placebo	Caffeine	ES
Jump height (imp-mom) [cm]	32.3 ± 7.6	33.8 ± 8.0*	0.82	29.9 ± 8.2	31.4 ± 7.5*	0.63	9.3 ± 4.5	12.7 ± 8.3	0.56
Peak velocity [m/s]	2.6 ± 0.3	2.7 ± 0.3*	0.67	2.5 ± 0.3	2.6 ± 0.3*	0.65	1.9 ± 0.3	2.1 ± 0.5	0.62
Peak power (W)	4496.2 ± 1384.5	4617.2 ± 1471.9*	0.70	4548.9 ± 1450.4	4631.7 ± 1377.9	0.33	1496.2 ± 453.4	1753 ± 683.3	0.70

Data is shown as mean SD. (*) Significant differences between caffeine and placebo at p < 0.05.

**Figure 1.**
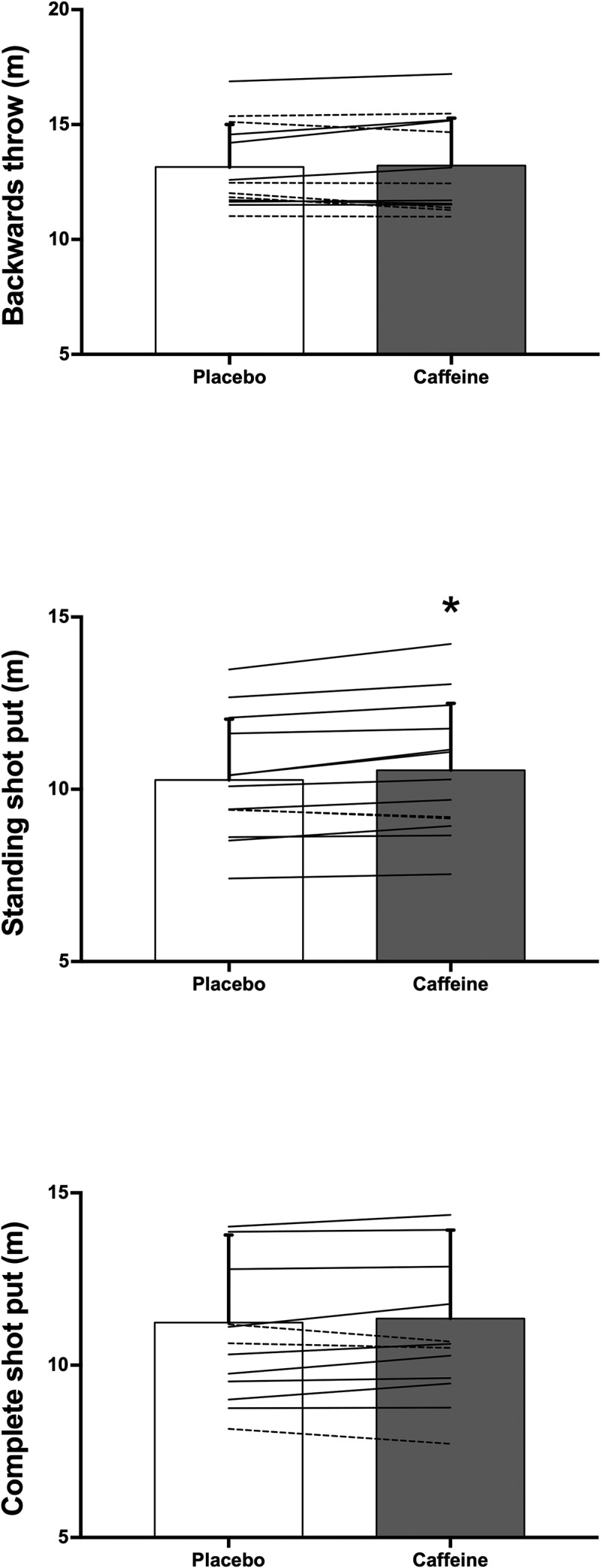
Individual responses for distance (m) during of the shot-put with different methods after the ingestion of 3 mg/kg/b.m. of caffeine or a placebo. Each line represents one individual from a sample of 13 individuals; continuous lines depict individuals who increased the caffeine distance with respect to placebo and the dashed line depicts the individual with decreased the caffeine distance with respect to placebo. (*) Significant differences between caffeine and placebo at p < 0.050.

Figure 1 depicts individual distances obtained in the different throwing tests to facilitate a more comprehensive view of the effect of caffeine. Caffeine increased the distance obtained in the standing shot put over the placebo (10.55 ± 1.94 *vs*. 10.27 ± 1.77 m; +2.6%, ES = 0.87, p = 0.009). Out of 13 shot putters, 11 improved the distance obtained in the standing shot put with caffeine intake. However, there were no significant differences in the backwards throw (13.21 ± 2.07 *vs*. 13.15 ± 1.85 m; +0.3%, ES = 0.13, p = 0.638) or in the complete shot put (11.35 ± 2.57 *vs*. 11.24 ± 2.54 m; +1.0%, ES = 0.33, p = 0.263). Nevertheless, seven shot putters improved the distance obtained in the backwards throw with caffeine intake and nine shot putters improved the distance obtained in the complete shot put with respect to the distances obtained in the placebo trial.

Fouls were similar between conditions, with 10 shot put throws judged as fouls with caffeine and 7 with the placebo. Table 2 depicts the prevalence of side effects the hours after the ingestion of caffeine or the placebo. There was no difference between conditions for any side effects analyzed. Six out of 13 athletes failed to guess the correct order of the placebo-caffeine conditions (significant differences between perception and real ingestion, Cohen’s Kappa = 0.39; p = 0.047; Bang Index = 0.23 and 0.54 for caffeine and placebo condition, respectively), so the blind design was successful. Also, 10 athletes indicated greater self-perception of muscle power in the caffeine condition compared to the placebo condition (31.15%, p = 0.051).

**Table 2.** Prevalence of side-effects the hours after with the ingestion of caffeine or placebo. Data are % affirmative responses obtained from 10 trained athletes.

	Placebo (%)	Caffeine (%)	*p*
Nervousness	0.0	10.0	0.317
Gastrointestinal problems	10	20	0.317
Activeness	10	30	0.157
Irritable	10	0	0.317
Muscular pain	10	20	0.317
Headache	40	10	0.083
Increased urine production	10	10	1.000

Data is shown as % of affirmative responses. (*) Significant differences between caffeine and placebo at p < 0.05.

## 4. Discussion

The aim of this investigation was to determine the effect of a moderate dose of caffeine (3 mg/kg/b.m.) on muscular power and strength tests and on shot put performance in trained athletes. The main outcomes of this investigation indicate that, in comparison to a placebo, the intake of caffeine increased jump performance in CMJ and SJ (Table 1) and increased the distance obtained in a standing shot put (Figure 1). However, the enhancements with caffeine did not reach statistical significance for handgrip dynamometry, push-up performance, and for the distances obtained in the backwards throw and complete shot put. Thus, caffeine ingestion, at a dose of 3 mg/kg/b.m. improved several aspects of physical performance in trained shot putters but the potential ergogenic effect of caffeine was of small magnitude on distance in a complete shot put.

There is a gap in the literature on the effects of caffeine on shot put performance or in any other throwing event in Athletics. Only Bellar et al. [18] have investigated the ergogenicity of a low dose of 100 mg of caffeine, but the dose selected in the study was lower than the suggested minimal doses of 1.5 mg/kg/b.m [11]. and there was no normalization of caffeine dose per participant’s body mass. In our study, we used 3 mg/kg of caffeine according to actual evidence that suggests that moderate doses of caffeine, habitually from 3 to 6 mg/kg/b.m., are needed to effectively increase maximal muscle strength, power output, and strength-endurance [3,10,20]. In addition, shot putters in Bellar et al.’s investigation [18] only performed a standing shot put (habitually used as a training routine), limiting the extrapolation of their findings to shot put performance during a real competition because this type of throw lacks a flight phase. In our research, the effect of caffeine was assessed by taking into account the best attempt with the longest distance (and not mean distance over several attempts), mimicking the conditions of a shot put competition. Additionally, the current study includes both the standing and complete shot put to assess the potential effect of caffeine on throws habitually used during training and competition. For these reasons, the current experiment is innovative and more ecologically valid in terms of applicability to trained shot putters than previous investigations.

Interestingly, caffeine supplementation was effective to enhance standing shot put performance but the effect of caffeine on the complete shot put did not reach statistical significance. Previous literature suggests that changes from 0.9 to 1.5% in the distance obtained during shot put are worthwhile improvements in elite throwers [33]. In our experiment, the effect of caffeine on the standing shot put surpassed this threshold (+2.6%), while caffeine improved distance in the complete shot put by 1.0%. Additionally, performance improvements with caffeine were greater than minimum detectable change in the standing shot put (mean improvement = 0.18 m vs MDC = 0.10 m) and in the complete shot put (mean improvement = 0.11 m vs MDC = 0.09 m). It is important to note that in the recent 2020 Olympic Games hosted in Tokyo, the difference between the gold and silver medals was 2.8/3.8% for men/women and the difference between the silver and bronze medals was of 0.8/0.9%. Collectively, all this information suggests that oral caffeine intake produced a non-statistically significant improvement of 1% in the distance obtained in a complete shot put. Although this effect was of small magnitude, it may represent a meaningful advantage for shot put performance beyond its statistical significance.

One of the hypotheses explaining the lack of a statistically significant effect of caffeine on the complete shot put is the difficulty to transfer the enhanced physicality induced with caffeine to overall shot put performance due to the technical complexity of this throw. Previous studies with moderate doses of caffeine (between 3 and 6 mg/kg/b.m.) have found ergogenic effects in simpler throw tasks, such as seated medicine ball throwing [34], bench press throw [35], or handball throws [36]. Nevertheless, the shot put is a complex throwing event that includes a preparation phase, a flight phase (glide or turn), a delivery phase, and a final recovery phase. Performance in the flight and delivery phases mainly depends on the strength and power output, and the proper application of these to the shot is obtained through multifaceted technical movements [21,37]. The application of power over a full range of multiple joint movements requires properly timed coordination of acceleration of all body segments in a sequence of action in order to produce a maximum velocity of the throwing hand [21,37,38]. The increase of the speed of the previous phases of the throw could negatively affect movement coordination and performance during the delivery phase, so kinematics and kinetics within each phase of shot put are codependent [37]. This could explain the improvement in the standing shot put (in line with previous research using simpler strength and power tasks [18,34–36]) in absence of ergogenic effects on the more complex complete shot put. Future research must elucidate if repeated shot put training sessions with caffeine ingestion could improve competition performance by adjusting the motor pattern to these improved exercise capacities.

The results of the present study showed a clear ergogenic effect of caffeine on CMJ height by 5.0% (~1.6 cm) and on SJ height by 6.4% (~1.5 cm; Table 1). These results are consistent with other studies evaluating the effects of caffeine on jump height in trained athletes [39–41]. In particular, our study agrees with that of Bloms et al. [41] who found increases of 4.3% for CMJ height and 5.4% for SJ height. In our study, caffeine increased concentric peak velocity by 1.8% (~0.1 m/s) and peak power by 2.5% (~121.1 W) in the CMJ which may explain why shot putters also obtained better distance with caffeine in the standing shot put [26]. From a practical perspective, enhanced lower limb power performance may be a key factor for better overall shot put performance, as the body’s displacement in the flight phase of the throw is highly dependent of the power generated in the lower limbs [37,41]. Additionally, during the putting action, force is transferred to the shot by an explosive drive while the perfect mechanics during for this drive requires the sequential actions of leg extension, rotation of the trunk, and extension of the putting arm are essential to produce [21]. Thus, it seems that caffeine intake is a potent ergogenic aid to increase lower limb power performance in shot put athletes which may constitute an potential advantage for the throw.

Our sample included 8 men and 5 women, as one of the objectives was to provide a study sample that reflects the potential benefits of caffeine for male and female trained shot putters. Women were tested regardless of the phase of the menstrual cycle as it was unfeasible to organize the date of the experimental trials to produce that all women were in the same phase of the menstrual cycle. However, the authors believe that, although this is a limitation of the experiment, the study outcomes were minimally affected by the lack of normalization of participants’ menstrual cycle phase. This is because previous research has found that the ingestion of 3 mg/kg/b.m. of caffeine produces a similar benefit during all phases of the menstrual cycle [42,43]. Moreover, other studies found that eumenorrheic females have similar muscle strength and power performance during the different phases of the menstrual cycle [44,45]. Collectively, all this information suggests that the potential performance benefits derived from acute caffeine intake may equally present in women athletes, irrespective of the menstrual cycle phase.

The study design presented different limitations that should be considered when interpreting the utility of caffeine for shot putters. First, the assessment procedures consisted of seven tests performed in succession (handgrip, CMJ, SJ, push-up, backwards throw, standing shot put and complete shot put). Although all tests were short in duration and resting times were set between tests to allow recovery, the all-out nature of all testing and the number of tests may have induced some fatigue that could have dampened the ergogenic effect of caffeine in the later tests. Future studies to examine the acute effects of caffeine on shot put performance could test different physical abilities in several sessions, thus minimizing the effects of accumulated fatigue. Additionally, the assessment of the ergogenic effect of caffeine during the real shot put competition may be necessary as this is the most ecological context. Second, the study was carried out in trained shot putters and results may not transfer to other throwing events in Athletics, lower-level athletes, or the general population. Third, despite being trained shot putters, the technique could have varied between days due to the fact that it was not analyzed. Fourth, this study did not include blood samples and thus we were unable to determine if participants performed the testing with peak serum caffeine concentrations. Last of all, each individual had different habituation to caffeine (one naïve, three low, eight mild and one moderate consumer [46]). Nevertheless, a secondary analysis comparing groups (naïve/low vs mild/moderate) showed no difference in the ergogenic effects, so habitual caffeine intake did not affect the result of this study.

## 5. Conclusion

To our knowledge, this is the first study that measures the effect of acute caffeine intake on complete shot put performance with an individualized dose. The findings of this investigation indicate that the pre-exercise intake of 3 mg/kg/b.m. of caffeine increased shot putters’ physical performance during the squat jump and countermovement jump and enhanced the distance for the standing shot put. Nevertheless, the effect of caffeine improving the complete shot put performance did not reach statistical significance but revealed a potential practical effect to enhance the distance obtained by 1.0%.
